# Editorial: Exploring traditional functional fermented foods and beverages for alleviating malnutrition and diabetes

**DOI:** 10.3389/fnut.2023.1286052

**Published:** 2023-10-09

**Authors:** Gloria Aderonke Otunola, Israel Sunmola Afolabi

**Affiliations:** ^1^Medicinal Plants & Economic Development (MPED) Research Centre, Department of Botany, University of Fort Hare, Alice, South Africa; ^2^Department of Chemistry, Faculty of Science, National Open University of Nigeria, Abuja, Nigeria; ^3^Department of Biochemistry, Covenant University, Ota, Nigeria

**Keywords:** functional foods, malnutrition, diabetes, fermented foods, fermented beverages

## Introduction

The Research Topic, *Exploring traditional functional fermented foods and beverages for alleviating malnutrition and diabetes* is a detailed summary of an interdisciplinary emerging area of study.

Food has always been researched and used alongside pharmacological approaches to optimize human health. “Functional foods,” including fermented foods are especially known to have beneficial effects for human nutrition and health. The primary role of such foods is to provide the body with the required amount of essential nutrients such as proteins, carbohydrates, vitamins, fats, and oils needed for healthy survival. Functional foods may be conventional (natural, whole-food rich in vitamins, minerals, phytonutrients, antioxidants, and healthy fats); modified (fortified with additional vitamins, minerals, probiotics, or fiber) or edible plants that have traits purposely bred in purple or gold potatoes, vitamin-enriched cereals, etc. Fermented foods and beverages are rich reservoirs of beneficial microbes with important nutritive and therapeutic properties, which provide health benefits that co-exist with nutraceutical and medical approaches for the prevention, treatment/management of malnutrition and chronic metabolic diseases, particularly diabetes.

Malnutrition results from deficiency or overconsumption of nutrients, as a result of (i) undernutrition-not getting enough protein, calories, or micronutrients because of starvation; chronic diseases or acute disease or injury, which often leads to wasting (low weight-for-height), stunting (height-for-age), and underweight (low weight-for-age); (ii) over-nutrition-excess intake of certain nutrients (protein, calories, or fat), that usually results in overweight or obesity.

Diabetes mellitus is an endocrine disorder characterized by chronic hyperglycaemia, which affects humans as a result of defects in insulin secretion or resistance. The incidence of diabetes has increased globally, making it one of the greatest health threats of the 21^st^ century. Both malnutrition and diabetes can be managed or even reversed through a healthy diet supplied as functional foods, beverages or supplements along with regular exercise.

This Research Topic focused on collating and documenting research information regarding the use of traditional fermented foods in the management and treatment of malnutrition and diabetes. As editors of this Research Topic, it was our pleasure to review the fascinating articles and reviews within the field. In this editorial, we summarize the main findings and perspectives detailed within each of the accepted articles.

## Regular consumption of pickled vegetables and fermented bean curd reduces the risk of diabetes: a prospective cohort study

In a prospective cohort study, Cai et al. revealed that regular consumption of pickled vegetables and fermented bean curd reduces the risk of diabetes. The authors studied 6,640 subjects without diabetes and followed them up for a median period of 6.49 years. Of these, 714 were diagnosed with diabetes during the study. Using a regression model with multivariable adjustment, it was revealed that diabetes risk was significantly reduced by consumption of 0–0.5 kg/month of pickled vegetables (OR = 0.77, 95% CI: 0.63, 0.94), further reduced by consumption of >0.5 kg/month of pickled vegetables (OR = 0.37, 95% CI: 0.23, 0.60) when compared to no consumption (both *P*-trend < 0.001), while consumption of fermented bean curd also reduced diabetes risk (OR = 0.68, 95% CI: 0.55, 0.84). The authors concluded that regular consumption of pickled vegetables and/or fermented bean curd can reduce the long-term risk of diabetes.

## Deciphering the mechanism of jujube vinegar on hyperlipoidemia through gut microbiome based on 16S rRNA, BugBase analysis, and the stamp analysis of KEEG

Hyperlipidemia is common and partly responsible for the increased vascular disease in patients with diabetes mellitus. Duan and Li investigated the role of the gut microbiome in treating hyperlipidemia with jujube vinegar and whether the action of jujube vinegar is related to the regulation of the gut microbiome. The authors showed that Jujube vinegar reduced body weight by 19.92%, serum TC, TG, and LDL-C by 25.09, 26.83, and 11.66%, and increased HDL-C by 1.44 times. Gut microbiome analysis revealed that jujube vinegar increased the intestinal microbial ASV count by 13.46%, and the F/B (Firmicutes/Bacteroidota) ratio by 2.08-fold in high-fat diet mice, The KEGG analysis showed that jujube vinegar was predominantly reflected in the biological process of gene function and related to signal transduction pathways. These activities by Jujube vinegar are mediated by controlling the gut microbiome and enhancing antioxidant capacity.

## Evaluation of *in vitro* and *in vivo* Glycemic Index of common staples made from varieties of White Yam (*Dioscorea rotundata*)

Consumption of high Glycemic Index (GI) foods is a risk factor for increasing prevalence of diabetes mellitus (DM). Eyinla et al. used both *in vitro* and *in vivo* models to evaluate the Glycemic Index of four commonly consumed products prepared from five varieties of White Yam (*Dioscorea rotundata*). They showed that all the products across each variety exhibited high GI and that irrespective of variety, processing Yam into *Amala* released Rapidly Digestible Starch (RDS) fractions, estimated *in vitro* GI (eGI) and GI faster compared with pounded yam. However, they concluded that *Alumaco* white yam variety particularly showed favorable properties applicable to the dietary management of diabetes. Although they propose and recommend that more processing methods and genetic diversity may need to be explored. By implication, it may be suggested that the consumption of all the products from each yam variety assessed exhibits a high glycaemic index and may therefore considered a possible risk factor contributing to the high prevalence of diabetes in regions where they are consumed. There may be a need for a more careful selection of the particular yam variety, and their processing methods for consumption, which may serve as critical factors for transforming and harnessing their health-beneficial properties. Also, the identified products from yam varieties with high glycaemic index as well as digestible starch content may be favorably applicable to consumption in support of sports nutrition where the abundant calories generated can be easily dispensed due to the requisite metabolic activities. These same varieties may not be appropriate for those with sedentary lifestyles as a means of preventing a further surge in the prevalence of diabetes mellitus among the population.

## Fermented foods and cardiometabolic health: definitions, current evidence, and future perspectives

Unhealthy diets contribute to the increasing burden of non-communicable diseases such as cardiometabolic diseases (CMDs), cardiovascular diseases, and type II diabetes. In their review, Li et al. gave an overview of (i) definitions of fermented foods, (ii) types and qualities of fermented foods consumed in Europe and globally, (iii) possible mechanisms between the consumption of fermented foods and cardiometabolic health, (iv) current state of epidemiological evidence on fermented food intake and cardiometabolic health, and (v) future perspectives and opportunities for improving the role of fermented foods in human diets.

## Author contributions

GO: Conceptualization, Writing—original draft, Writing—review and editing. IA: Writing—original draft, Writing—review and editing.

